# 4-Isopropyl­amino-3-nitro­benzonitrile

**DOI:** 10.1107/S1600536811051737

**Published:** 2011-12-21

**Authors:** Hong-sheng Jia

**Affiliations:** a Department of Biological and Chemical Engineering, Chien-shiung Institute of Technology, Taicang 215411, Suzhou, People’s Republic of China

## Abstract

In the title compound, C_10_H_11_N_3_O_2_, the nitro group is essentially coplanar with the aromatic ring [dihedral angle = 3.4 (3)°] and forms an intra­molecular N—H⋯O hydrogen bond with the amine group. In the crystal, weak aromatic C—H⋯O and C—H⋯N hydrogen bonds link the mol­ecules. Weak aromatic ring π–π inter­actions [minimum ring centroid–centroid separation = 3.9841 (16) Å] are also present.

## Related literature

For the synthesis of the title compound, see: Ates-Alagoz & Buyukbingol (2001 ▶). For bond-length data, see: Allen *et al.* (1987 ▶).
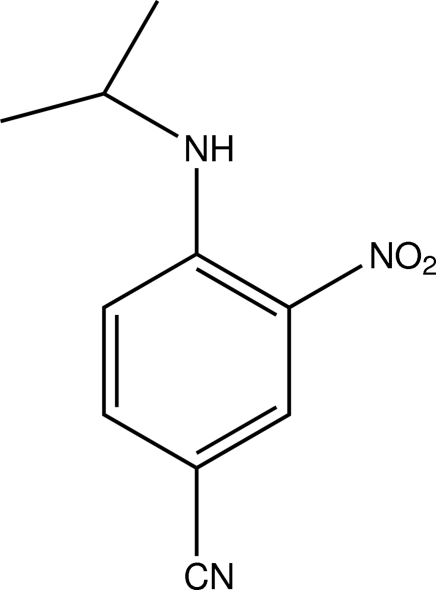


## Experimental

### 

#### Crystal data

C_10_H_11_N_3_O_2_
*M* *_r_* = 205.22
Monoclinic, 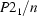

*a* = 6.6640 (13) Å
*b* = 20.678 (4) Å
*c* = 7.8900 (16) Å
β = 105.74 (3)°
*V* = 1046.5 (4) Å^3^
*Z* = 4
Mo *K*α radiation
μ = 0.09 mm^−1^
*T* = 293 K
0.20 × 0.10 × 0.10 mm

#### Data collection

Enraf–Nonius CAD-4 four-circle diffractometer
Absorption correction: ψ scan (North *et al.*, 1968 ▶) *T*_min_ = 0.982, *T*_max_ = 0.991
2821 measured reflections
1926 independent reflections
1228 reflections with *I* > 2σ(*I*)
*R*_int_ = 0.020
3 standard reflections every 200 reflections  intensity decay: 1%

#### Refinement

*R*[*F* ^2^ > 2σ(*F* ^2^)] = 0.050
*wR*(*F* ^2^) = 0.170
*S* = 1.00
1926 reflections
137 parameters
H-atom parameters constrained
Δρ_max_ = 0.20 e Å^−3^
Δρ_min_ = −0.17 e Å^−3^

### 

Data collection: *CAD-4 Software* (Enraf–Nonius, 1994) ▶; cell refinement: *CAD-4 Software*; data reduction: *XCAD4* (Harms & Wocadlo, 1995 ▶); program(s) used to solve structure: *SHELXS97* (Sheldrick, 2008 ▶); program(s) used to refine structure: *SHELXL97* (Sheldrick, 2008 ▶); molecular graphics: *PLATON* (Spek, 2009 ▶); software used to prepare material for publication: *SHELXL97*.

## Supplementary Material

Crystal structure: contains datablock(s) global, I. DOI: 10.1107/S1600536811051737/zs2168sup1.cif

Structure factors: contains datablock(s) I. DOI: 10.1107/S1600536811051737/zs2168Isup2.hkl

Supplementary material file. DOI: 10.1107/S1600536811051737/zs2168Isup3.cml

Additional supplementary materials:  crystallographic information; 3D view; checkCIF report

## Figures and Tables

**Table 1:** Hydrogen-bond geometry (Å, °)

*D*—H⋯*A*	*D*—H	H⋯*A*	*D*⋯*A*	*D*—H⋯*A*
N3—H3*A*⋯O2	0.86	1.99	2.643 (2)	132
C1—H1*A*⋯N1^i^	0.93	2.61	3.469 (3)	153
C4—H4*A*⋯O1^ii^	0.93	2.40	3.298 (3)	163
C5—H5*A*⋯N1^iii^	0.93	2.60	3.529 (4)	175

Symmetry codes: (i) 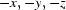; (ii) 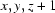; (iii) 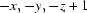.

**Fig. 1.**
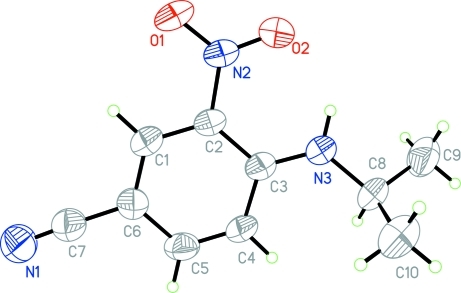
The molecular structure of the title compound showing the atom-numbering scheme, with displacement ellipsoids drawn at the 30% probability level.

**Fig. 2.**
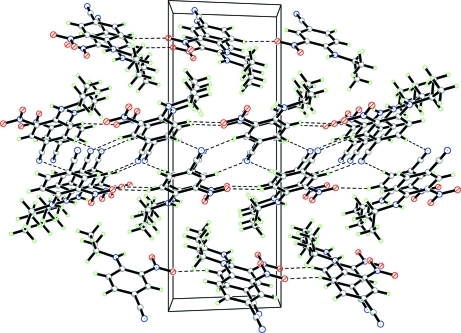
A packing diagram of the title compound, with intermolecular hydrogen bonds shown as dashed lines.

## supplementary crystallographic information

### Comment

We report herein the crystal structure of the title compound C_10_H_11_N_3_O_2_. In this molecule (Fig. 1), the bond lengths and angles (Allen *et al.*, 1987) are within normal ranges. The nitro group is essentially coplanar with the aromatic ring forming a dihedral angle of 3.4 (3)° with the ring. The amine H atom forms an intramolecular hydrogen bond with a nitro O-atom acceptor (O2) (Table 1). In the crystal structure, intermolecular aromatic C—H···O and C—H···N hydrogen bonds link the molecules (Fig. 2) while also present are weak aromatic ring π–π interactions [minimum ring centroid separation, 3.9841 (16) Å].

### Experimental

The title compound was synthesized using the procedure of (Ates-Alagoz & Buyukbingol, 2001). 4-Chloro-3-nitrobenzonitrile (4.2 g, 0.023 mol) was refluxed in 25 ml of *t*-propylamine and 50 ml of tetrahydrofuran for 4 h. The solvent was then evaporated and water was added to give a precipitate which was collected by filtration and washed with cold ethanol (2 × 15 ml) to afford the yellow solid (4.2 g, 89%). The pure title compound was obtained by crystallizing from ethanol, with crystals suitable for X-ray diffraction obtained by slow room-temperature evaporation of an ethanol solution.

### Refinement

Hydrogen atoms were positioned geometrically, with C—H = 0.93 Å (aromatic), 0.97 Å (methylene) or 0.96 Å (methyl) and N—H = 0.86 Å, an were allowed to ride on their parent atoms, with *U*_iso_(H) = 1.2*U*_eq_(N, aromatic or methylene C) or 1.5*U*_eq_(methyl C).

### Crystal data

**Table:**

C_10_H_11_N_3_O_2_	*F*(000) = 432
*M**_r_* = 205.22	*D*_x_ = 1.303 Mg m^−^^3^
Monoclinic, *P*2_1_/*n*	Mo *K*α radiation, λ = 0.71073 Å
Hall symbol: -P 2yn	Cell parameters from 25 reflections
*a* = 6.6640 (13) Å	θ = 9–13°
*b* = 20.678 (4) Å	µ = 0.09 mm^−^^1^
*c* = 7.8900 (16) Å	*T* = 293 K
β = 105.74 (3)°	Block, yellow
*V* = 1046.5 (4) Å^3^	0.20 × 0.10 × 0.10 mm
*Z* = 4	

### Data collection

**Table:**

Enraf–Nonius CAD-4 four-circle diffractometer	1228 reflections with *I* > 2σ(*I*)
Radiation source: fine-focus sealed tube	*R*_int_ = 0.020
graphite	θ_max_ = 25.4°, θ_min_ = 2.0°
ω/2θ scans	*h* = 0→8
Absorption correction: ψ scan (North *et al.*, 1968)	*k* = −6→24
*T*_min_ = 0.982, *T*_max_ = 0.991	*l* = −9→9
2821 measured reflections	3 standard reflections every 200 reflections
1926 independent reflections	intensity decay: 1%

### Refinement

**Table:**

Refinement on *F*^2^	Secondary atom site location: difference Fourier map
Least-squares matrix: full	Hydrogen site location: inferred from neighbouring sites
*R*[*F*^2^ > 2σ(*F*^2^)] = 0.050	H-atom parameters constrained
*wR*(*F*^2^) = 0.170	*w* = 1/[σ^2^(*F*_o_^2^) + (0.10*P*)^2^ + 0.040*P*] where *P* = (*F*_o_^2^ + 2*F*_c_^2^)/3
*S* = 1.00	(Δ/σ)_max_ < 0.001
1926 reflections	Δρ_max_ = 0.20 e Å^−^^3^
137 parameters	Δρ_min_ = −0.17 e Å^−^^3^
0 restraints	Extinction correction: *SHELXL97* (Sheldrick, 2008), Fc^*^=kFc[1+0.001xFc^2^λ^3^/sin(2θ)]^-1/4^
Primary atom site location: structure-invariant direct methods	Extinction coefficient: 0.038 (8)

### Special details

**Table:**

Geometry. All e.s.d.'s (except the e.s.d. in the dihedral angle between two l.s. planes) are estimated using the full covariance matrix. The cell e.s.d.'s are taken into account individually in the estimation of e.s.d.'s in distances, angles and torsion angles; correlations between e.s.d.'s in cell parameters are only used when they are defined by crystal symmetry. An approximate (isotropic) treatment of cell e.s.d.'s is used for estimating e.s.d.'s involving l.s. planes.
Refinement. Refinement of *F*^2^ against ALL reflections. The weighted *R*-factor *wR* and goodness of fit *S* are based on *F*^2^, conventional *R*-factors *R* are based on *F*, with *F* set to zero for negative *F*^2^. The threshold expression of *F*^2^ > σ(*F*^2^) is used only for calculating *R*-factors(gt) *etc*. and is not relevant to the choice of reflections for refinement. *R*-factors based on *F*^2^ are statistically about twice as large as those based on *F*, and *R*- factors based on ALL data will be even larger.

### Fractional atomic coordinates and isotropic or equivalent isotropic displacement parameters (Å^2^)

**Table:**

	*x*	*y*	*z*	*U*_iso_*/*U*_eq_	
C1	0.3856 (4)	0.06884 (12)	0.1995 (3)	0.0484 (6)	
H1A	0.3429	0.0582	0.0805	0.058*	
O1	0.6054 (3)	0.10761 (14)	−0.0153 (2)	0.1029 (10)	
N1	−0.0699 (4)	−0.01652 (13)	0.2024 (3)	0.0764 (8)	
O2	0.8456 (3)	0.15107 (10)	0.1884 (2)	0.0683 (6)	
N2	0.6789 (3)	0.12211 (12)	0.1391 (2)	0.0578 (6)	
C2	0.5671 (3)	0.10375 (11)	0.2650 (3)	0.0440 (6)	
N3	0.8123 (3)	0.15551 (10)	0.5144 (2)	0.0526 (6)	
H3A	0.8880	0.1652	0.4454	0.063*	
C3	0.6395 (3)	0.12144 (11)	0.4461 (3)	0.0427 (6)	
C4	0.5119 (4)	0.10035 (12)	0.5527 (3)	0.0505 (7)	
H4A	0.5516	0.1107	0.6719	0.061*	
C5	0.3355 (4)	0.06600 (12)	0.4889 (3)	0.0532 (7)	
H5A	0.2576	0.0529	0.5644	0.064*	
C6	0.2680 (3)	0.04974 (12)	0.3092 (3)	0.0490 (6)	
C7	0.0803 (4)	0.01312 (14)	0.2466 (3)	0.0582 (7)	
C8	0.8847 (4)	0.17789 (13)	0.6982 (3)	0.0544 (7)	
H8A	0.8699	0.1422	0.7757	0.065*	
C9	1.1128 (5)	0.19404 (18)	0.7342 (4)	0.0861 (10)	
H9A	1.1873	0.1568	0.7113	0.129*	
H9B	1.1666	0.2067	0.8551	0.129*	
H9C	1.1300	0.2289	0.6591	0.129*	
C10	0.7585 (5)	0.23444 (15)	0.7327 (4)	0.0748 (9)	
H10A	0.6149	0.2219	0.7096	0.112*	
H10B	0.7704	0.2697	0.6570	0.112*	
H10C	0.8098	0.2476	0.8533	0.112*	

### Atomic displacement parameters (Å^2^)

**Table:**

	*U*^11^	*U*^22^	*U*^33^	*U*^12^	*U*^13^	*U*^23^
C1	0.0550 (14)	0.0553 (15)	0.0343 (11)	0.0006 (12)	0.0111 (10)	0.0007 (10)
O1	0.1008 (16)	0.178 (3)	0.0316 (10)	−0.0490 (16)	0.0207 (10)	−0.0053 (13)
N1	0.0729 (16)	0.0899 (19)	0.0677 (15)	−0.0259 (15)	0.0211 (12)	−0.0044 (14)
O2	0.0723 (13)	0.0866 (14)	0.0533 (10)	−0.0266 (11)	0.0294 (9)	−0.0077 (10)
N2	0.0632 (14)	0.0774 (16)	0.0359 (11)	−0.0117 (12)	0.0185 (9)	0.0019 (10)
C2	0.0502 (13)	0.0511 (14)	0.0323 (11)	−0.0016 (11)	0.0137 (9)	0.0039 (10)
N3	0.0573 (12)	0.0644 (13)	0.0384 (10)	−0.0135 (11)	0.0168 (9)	−0.0083 (9)
C3	0.0488 (13)	0.0452 (13)	0.0349 (11)	0.0013 (11)	0.0128 (10)	0.0009 (10)
C4	0.0603 (15)	0.0625 (16)	0.0313 (11)	−0.0063 (13)	0.0167 (10)	−0.0043 (11)
C5	0.0613 (16)	0.0611 (16)	0.0434 (13)	−0.0030 (13)	0.0245 (11)	0.0034 (11)
C6	0.0484 (14)	0.0523 (15)	0.0464 (13)	−0.0027 (12)	0.0133 (11)	0.0008 (11)
C7	0.0610 (16)	0.0653 (17)	0.0497 (14)	−0.0064 (15)	0.0174 (12)	0.0020 (13)
C8	0.0634 (16)	0.0613 (16)	0.0371 (12)	−0.0052 (13)	0.0112 (11)	−0.0126 (11)
C9	0.073 (2)	0.109 (3)	0.074 (2)	−0.0228 (19)	0.0156 (16)	−0.0385 (19)
C10	0.090 (2)	0.072 (2)	0.0614 (16)	0.0019 (17)	0.0199 (15)	−0.0147 (15)

### Geometric parameters (Å, °)

**Table:**

C1—C6	1.374 (3)	C4—H4A	0.9300
C1—C2	1.383 (3)	C5—C6	1.407 (3)
C1—H1A	0.9300	C5—H5A	0.9300
O1—N2	1.221 (2)	C6—C7	1.430 (4)
N1—C7	1.144 (3)	C8—C9	1.506 (4)
O2—N2	1.229 (3)	C8—C10	1.507 (4)
N2—C2	1.445 (3)	C8—H8A	0.9800
C2—C3	1.426 (3)	C9—H9A	0.9600
N3—C3	1.333 (3)	C9—H9B	0.9600
N3—C8	1.473 (3)	C9—H9C	0.9600
N3—H3A	0.8600	C10—H10A	0.9600
C3—C4	1.417 (3)	C10—H10B	0.9600
C4—C5	1.349 (3)	C10—H10C	0.9600
			
C6—C1—C2	120.3 (2)	C1—C6—C7	122.0 (2)
C6—C1—H1A	119.8	C5—C6—C7	119.0 (2)
C2—C1—H1A	119.8	N1—C7—C6	177.7 (3)
O1—N2—O2	121.3 (2)	N3—C8—C9	107.4 (2)
O1—N2—C2	118.7 (2)	N3—C8—C10	111.8 (2)
O2—N2—C2	120.05 (18)	C9—C8—C10	112.2 (2)
C1—C2—C3	122.2 (2)	N3—C8—H8A	108.4
C1—C2—N2	116.20 (19)	C9—C8—H8A	108.4
C3—C2—N2	121.6 (2)	C10—C8—H8A	108.4
C3—N3—C8	125.47 (19)	C8—C9—H9A	109.5
C3—N3—H3A	117.3	C8—C9—H9B	109.5
C8—N3—H3A	117.3	H9A—C9—H9B	109.5
N3—C3—C4	120.94 (19)	C8—C9—H9C	109.5
N3—C3—C2	124.1 (2)	H9A—C9—H9C	109.5
C4—C3—C2	114.9 (2)	H9B—C9—H9C	109.5
C5—C4—C3	122.9 (2)	C8—C10—H10A	109.5
C5—C4—H4A	118.6	C8—C10—H10B	109.5
C3—C4—H4A	118.6	H10A—C10—H10B	109.5
C4—C5—C6	120.7 (2)	C8—C10—H10C	109.5
C4—C5—H5A	119.7	H10A—C10—H10C	109.5
C6—C5—H5A	119.7	H10B—C10—H10C	109.5
C1—C6—C5	119.0 (2)		
			
C6—C1—C2—C3	−0.2 (4)	N2—C2—C3—C4	179.5 (2)
C6—C1—C2—N2	−179.4 (2)	N3—C3—C4—C5	179.8 (2)
O1—N2—C2—C1	1.9 (4)	C2—C3—C4—C5	0.1 (4)
O2—N2—C2—C1	−177.3 (2)	C3—C4—C5—C6	−0.6 (4)
O1—N2—C2—C3	−177.4 (3)	C2—C1—C6—C5	−0.3 (4)
O2—N2—C2—C3	3.5 (4)	C2—C1—C6—C7	−179.4 (2)
C8—N3—C3—C4	−3.4 (4)	C4—C5—C6—C1	0.6 (4)
C8—N3—C3—C2	176.1 (2)	C4—C5—C6—C7	179.8 (2)
C1—C2—C3—N3	−179.4 (2)	C3—N3—C8—C9	161.5 (3)
N2—C2—C3—N3	−0.1 (4)	C3—N3—C8—C10	−75.0 (3)
C1—C2—C3—C4	0.2 (3)		

### Hydrogen-bond geometry (Å, °)

**Table:**

*D*—H···*A*	*D*—H	H···*A*	*D*···*A*	*D*—H···*A*
N3—H3A···O2	0.86	1.99	2.643 (2)	132.
C1—H1A···N1^i^	0.93	2.61	3.469 (3)	153.
C4—H4A···O1^ii^	0.93	2.40	3.298 (3)	163.
C5—H5A···N1^iii^	0.93	2.60	3.529 (4)	175.

Symmetry codes: (i) −*x*, −*y*, −*z*; (ii) *x*, *y*, *z*+1; (iii) −*x*, −*y*, −*z*+1.
